# Characterization of novel neutralizing mouse monoclonal antibody JM1-24-3 developed against MUC18 in metastatic melanoma

**DOI:** 10.1186/s13046-020-01722-8

**Published:** 2020-12-05

**Authors:** Runhua Feng, Yuling Wang, Vijaya Ramachandran, Qinhong Ma, Matthew M. May, Ming Li, Joe X. Zhou, Xiang Xu, Kejing Xu, Shenying Fang, Weiya Xia, Dawen Sui, Huey Liu, Xiaolian Gao, Victor Prieto, Stephen C. Blacklow, Mason Lu, Jeffrey E. Lee

**Affiliations:** 1 Department of Surgical Oncology, The University of Texas MD Anderson Cancer Center, Houston, TX 77030 USA; 2 Department of General Surgery, Ruijin Hospital, Shanghai Jiao Tong University School of Medicine, Shanghai, 200025 China; 3 MedAbome, Inc., Fremont, CA 94538 USA; 4 Department of Otolaryngology, Mayo School of Graduate Medical Education, Rochester, MN 55905 USA; 5 Department of Pathology, Anhui Province Hospital, Anhui Medical University, Hefei, Anhui 230032 China; 6 LC Sciences, LLC, Houston, TX 77054 USA; 7 Department of Biological Chemistry and Molecular Pharmacology, Harvard University School of Medicine, Boston, MA 02115 USA; 8 Department of Pathology, UT Southwestern Medical Center, Dallas, TX 75390 USA; 9 Department of Molecular & Cellular Oncology, The University of Texas MD Anderson Cancer Center, Houston, TX 77030 USA; 10 Department of Biostatistics, The University of Texas MD Anderson Cancer Center, Houston, TX 77030 USA; 11 Department of Breast Medical Oncology, The University of Texas MD Anderson Cancer Center, Houston, TX 77030 USA; 12 Department of Biology and Biochemistry, The University of Houston, Houston, TX 77204 USA; 13 Department of Pathology, The University of Texas MD Anderson Cancer Center, Houston, TX 77030 USA

**Keywords:** MUC18, CD146, Metastatic melanoma, Therapeutic antibody, Targeted therapy

## Abstract

**Background:**

MUC18 is a glycoprotein highly expressed on the surface of melanoma and other cancers which promotes tumor progression and metastasis. However, its mechanism of action and suitability as a therapeutic target are unknown.

**Methods:**

A monoclonal antibody (mAb) (JM1-24-3) was generated from metastatic melanoma tumor live cell immunization, and high-throughput screening identified MUC18 as the target.

**Results:**

Analysis of molecular interactions between MUC18 and JM1-24-3 revealed that the downstream signaling events depended on binding of the mAb to a conformational epitope on the extracellular domain of MUC18. JM1-24-3 inhibited melanoma cell proliferation, migration and invasion in vitro and reduced tumor growth and metastasis in vivo.

**Conclusion:**

These results confirm that MUC18 is mechanistically important in melanoma growth and metastasis, suggest that the MUC18 epitope identified is a promising therapeutic target, and that the JM1-24-3 mAb may serve as the basis for a potential therapeutic agent.

## Background

Melanoma is the most lethal form of common skin cancers. While targeted and immune-based therapies are increasingly promising, not all patients benefit, thus establishing the need to identify novel targets that can contribute to improved therapeutic strategies. Using live melanoma cell immunization and high-throughput screening (HTS) [1], we generated a novel neutralizing monoclonal antibody (mAb) directed against a melanoma cell-surface antigen, which we subsequently identified as the functional, glycosylated protein MUC18.

MUC18, also known as MCAM (melanoma cell adhesion molecule), CD146 (cluster of differentiation 146), or METCAM/MelCAM (metastatic melanoma CAM), is expressed on the surface of metastatic melanoma and other cancer cells [2]. Expression of MUC18 has been demonstrated to promote tumorigenesis and tumor progression, and therapeutic targeting of MUC18 can reduce bone metastasis in a prostate cancer model [3]. Investigations outlined here demonstrated that the mAb developed was capable of specifically interacting with MUC18 on melanoma cells, initiating downstream signaling events that were associated with inhibition of melanoma cell proliferation, migration, and invasion in vitro*,* and reduction in tumor growth and metastasis in vivo. Furthermore, we found that these signaling events depended on binding of the mAb to a conformational epitope on the extracellular domain of MUC18.

## Materials and methods

### Animal study

A/J mice (6–8 weeks old, male) (Harlan Sprague Dawley, Inc., Indianapolis, IN) and nu/nu mice (4–8 weeks old, male) (JAX, Bar Harbor, Maine) were used for this study. Mice were maintained and experiments were performed under protocols (IACUC00001239-RN00 and IACUC00000731-RN01) approved by the Institutional Animal Care and Use Committee (IACUC).

### Cell lines & tissues & other resources

A375 cells (primary cell line with low metastatic capacity) [4]; and A2058 and WM266-4 cells (highly metastatic melanoma cells), SP2/0 (mouse myeloma cells), EC-RF24 (immortalized human endothelial cells) were purchased from American Type Culture Collection (ATCC, Manassas, VA). Human peripheral blood mononuclear cells (PBMCs) were separated by using Ficoll-Plaque Plus (Ficoll, GE Healthcare Biosciences) from healthy donor blood (Gulf Coast Regional Blood Center). Other cell lines were gifts from collaborators. Cell lines were grown in serum free medium MD6 derived from Dulbecco’s modified Eagle’s medium (DMEM, Gibco) supplemented with 5% FBS and 1% penicillin-streptomycin. Formalin-fixed paraffin embedded (FFPE) tissue slides and tissue microarrays (TMAs) were prepared from a variety of tumors and normal tissues from our institutional tissue bank under institutional review board protocols (LAB09–0197 and PA11–0957). Tunicamycin (Cat. #T7765, Sigma-Aldrich, MO) and Dynabeads conjugated with Protein A (Cat. #14311D, Invitrogen, Inc., Carlsbad, CA) were also purchased.

### Antigen & Antibodies

Human recombinant protein CD146 (MCAM) was purchased from OriGene Technologies (Cat. #TP308937, Rockville, MD). Commercial antibodies used in this study include anti-human MUC18 (mouse mAb, Cat. #MAB932; goat polyclonal Ab, Cat. #AF932, R&D Systems, Inc., Minneapolis, MN; mouse mAb, Cat # ab233923; AbCam, Cambridge, MA) and an irrelevant mAb (served as isotype control antibody) and goat serum (IgG). FITC, HRP and Alexa fluor 647 conjugated goat anti-mouse IgG (Cat. #115–095-071, 115–035-071, 115–606-062, Jackson ImmunoResearch Lab, West Grove, PA) served as secondary antibodies.

### Live-cell immunization

Five million each of A375, A2058 and WM266-4 metastatic melanoma cells were injected subcutaneously (s.c.) into three A/J mice every 2 weeks × 3, followed by an intraperitoneal (i.p.) boost. Three days after boost, spleen cells from the immunized mice were collected and fused with myeloma SP2/0 cells to generate hybridomas [5].

### High-throughput screening (HTS) using fluorescence-activated cell sorting (FACS)

The BD LSR II Flow Cytometry System with HTS autosampler (Becton Dickinson) was used to screen for mAbs secreted from the hybridomas that bound to the mixed melanoma cell lines as outlined previously [1]. Mean fluorescence intensity (MFI) and the percentage of the stained cell peaks from screening and counter-screening plates were determined using the BD LSR II FACS-HTS system.

### Glycosylation and Lectin binding analyses

FACS analysis was used to detect the carbohydrate group of the MUC18 glycoprotein from cells (1 × 10^5^) individually seeded in flasks with or without conditioned medium containing 3.0 μg/mL tunicamycin in DMSO (Dimethyl sulfoxide) and cultured for 24 h. Cells were then harvested and stained with JM1-24-3 or an irrelevant mAb for quantification of MFI. Cell lysates were probed with JM1-24-3 and β-actin mAb in WB (western blot). FLISA (Fluorescence-linked immunosorbent assay) was used for SNL (*Sambucus nigra* lectin) binding analysis, in which individual cell lysates were coated on plates and incubated with SNL-FITC (Fluorescein isothiocyanate) which was then competed by serial dilution of JM1-24-3, starting with 20 μg/mL, with or without SNL. Similar FLISA assays were conducted for other lectins [DSA (*Datura stramonium* agglutinin) lectin and DSA-FITC; LCA (*Lens culinaris* agglutinin) lectin and LCA-FITC; WGA (Wheat germ agglutinin) lectin and WGA-FITC].

### Epitope mapping

The biochip was spotted in triplicate with MUC18 8-mer and 6-mer oligopeptides at one amino acid resolution (acetyl capped at the N-termini on the ChipMDA_130046) and then incubated with JM1-24-3 (1.0 μg/mL) at 4 °C for 2 h, followed by washing and incubation with goat anti-mouse IgGFc Alexa 647 conjugated (0.01 μg/mL) at 4 °C for 2 h, and then washed again. The image of the biochip was scanned in Cy5 channel and data analyzed.

### Immunohistochemistry (IHC) analysis

The expression of MUC18 on melanoma patient tissues and on TMA containing several normal tissues and cancers was detected by IHC using JM1-24-3 mAb (1:1000) as described previously [1].

### Immunoprecipitation (IP) and mass spectrometry (MS)

IP was conducted on cell lysates (100 μg) using Protein A beads (0.2 mL) and JM1-24-3 (10 μg) or irrelevant control antibodies as described previously [1]. Excised bands were analyzed with a ProteinChip system in Series 4000 Mass Spectrometry (Bio-Rad) as described previously [1].

### Reverse phase protein Array (RPPA)

WM266-4 cells were treated with JM1-24-3 or its F (ab’)_2_ fragment for 1 h or 6 h and RPPA conducted [6]. Heat map results were analyzed for changes in protein expression using Ingenuity Pathway Analysis (IPA) [7] software (Qiagen Bioinformatics) to identify down-stream signaling pathways.

### Structural modeling

The homology model of the extracellular domain (residues 5–559) of MUC18 was generated using the Swiss Model web interface [8], using the functional motif of “search for templates”. The first approach modes were used to generate an initial homology model and accompanying sequence alignment, which was then manually modified using Coot [9]. A final correction of the alignment was then performed in Swiss Pdb Viewer. This model and the accompanying corrected sequence alignment were then input into the Optimization Mode of Swiss Model. The final pdb files were used to display the overall conformation and the antibody epitope locations.

The homology model of JM1-24-3 variable domain was generated using BioLuminate v.1.1 (Schrödinger, New York, NY). Template coordinates for heavy chain and light chain were chosen from PDB structure 4BKL and 6I1O respectively based on the sequence homology search. CDRs were modeled using the BioLuminate Basic Loop Model function. Experimental data were incorporated into the program for interaction. All antibody structure images were generated using Maestro and PyMol (Schrödinger, New York, NY).

### In vitro cell proliferation, migration and invasion studies

These studies were conducted on incubating cells with and without JM1-24-3 or irrelevant mAb (150 μg/mL) for 1 week as described previously [1]. For migration assays, 3 × 10^4^ cells in serum-free medium (300 μL) were incubated with and without JM1-24-3 (150 μg/mL) or irrelevant mAb for 24 h. Invasion assays were carried out by a similar protocol using QCM ECMatrix cell Invasion Assay Kit (EMD Millipore).

### In vivo tumor Xenograft studies of melanoma growth and metastasis

Athymic nude (*nu/nu*) mice were injected subcutaneously with WM266-4 cells (1 million/0.1 mL). After 5 days mice were randomly divided into two groups and treated with either JM1-24-3 (6 mg/kg body weight/i.p./twice a week) (*n* = 11) or with the same dose of irrelevant mAb (*n* = 8) for 45 days. Tumor volume was measured with calipers every 4 days. At the end of the experiment, tumors were excised and weighed. Mice body weight was measured every 4 days and any reduction > 10% from initial weight was considered as toxicity. For metastasis studies, mice were pre-treated 1 day before with JM1-24-3 (*n* = 5) or with irrelevant mAb (*n* = 7), following which all received a tail vein injection with WM266-4 cells (1 million); mAb treatment was administered every 4 days. All mice were sacrificed at day 45 and lungs were harvested for H&E staining.

### Statistical analysis

Experiments were repeated at least in replicate and data expressed as mean ± standard deviation (SD). Representative figures are presented for FACS, ELISA, FLISA and MS analysis. Differences were analyzed with a two-tailed Student’s t-test or Wilcoxon rank-sum test and paired t-test for serial dilution studies. *P* values < 0.05 were considered as statistically significant.

## Results

### Screening and identification of anti-MUC18 mAb

#### FACS-HTS of Hybridomas showed differential binding of JM1-24-3 with low and high metastatic melanoma cells

To obtain mAbs against the conformational epitopes of cell surface antigens, we used a strategy of live-cell immunization using a mixture of three melanoma cell lines, including A375 (primary cell line with low metastatic capacity), A2058 (highly metastatic cell line from lymph node), and WM266-4 (highly metastatic cell line from lymph node) as immunogens.

**Fig. 1:**
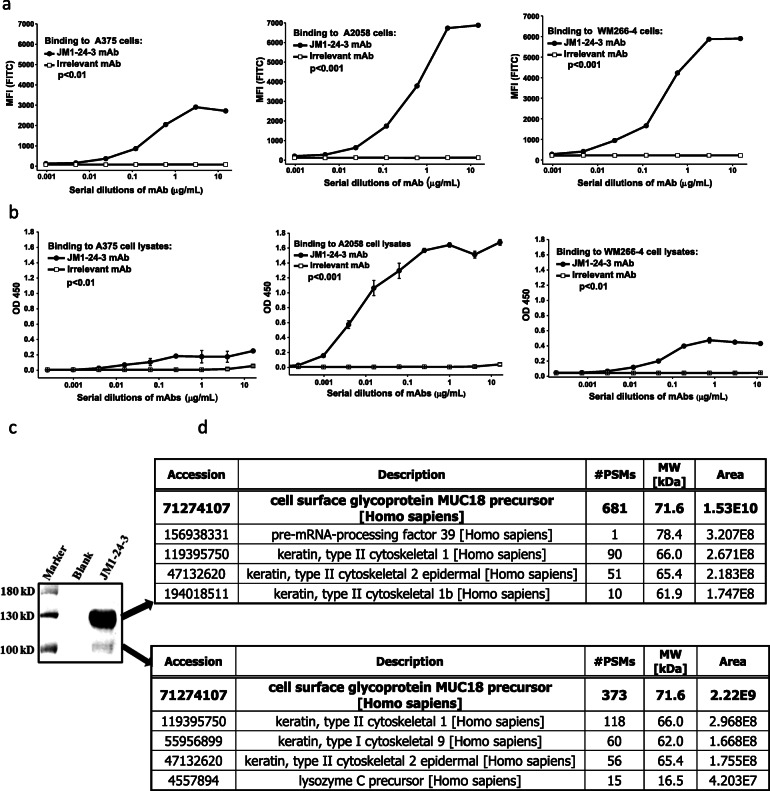
Screening and identification of anti-MUC18 mAb. *Binding of JM1-24-3 to its target on melanoma cell surface is verified by FACS and ELISA* **a** FACS analysis of JM1-24-3 binding on the surfaces of live melanoma cells showed that high metastatic cell lines A2058 and WM266-4 had significantly higher binding compared to low metastatic cell line A375. **b** ELISA analysis of JM1-24-3 binding with the immobilized melanoma cell lysates showed similar binding pattern as of FACS. High metastatic cell line A2058 showed the maximum binding. *MUC18 protein is defined as the target of JM1-24-3 by immunoprecipitation (IP) and mass spectrometry (MS) analyses* **c** Immunoprecipitation of WM266-4 lysates with JM1-24-3 showed two bands - 135kD (high intensity) and 110kD (faint band) by SDS-PAGE Coomassie blue staining. **d** MS analysis showed that MUC18 glycoprotein had invariably the highest score among all the hits for both the bands

By FACS-HTS, hybridoma supernatant (the hybridoma colony number is about 23,000) binding with the three melanoma cell lines versus normal human PBMC cells was assessed (data not shown). Of the supernatants tested, less than 5% demonstrated binding to the immunization cell lines but no binding to the human PBMC cells. Based on stronger binding signals in FACS assay, 20 promising hybridoma candidates were selected and subcloned, and again tested for binding. Out of those 20 candidates, given its strong binding as measured by FACS assay, JM1-24-3 hybridoma clone was chosen for further evaluation and validation. JM1-24-3 hybridoma was further subcloned, and secreted mAb was purified and identified as an IgG1 heavy chain and κ light chain antibody by mouse antibody isotyping [1]. FACS analysis of binding of JM1-24-3 to each of the immunization cell lines was done (Fig. 1a). Highly metastatic cell lines WM266-4 and A2058 showed greater binding in terms of MFI compared to low metastatic cell line A375 (*p* < 0.01). Similar relative binding patterns were seen with ELISA. Highly metastatic cell line A2058 demonstrated maximum binding (1.67 ± 0.04) at 1.0 μg/mL optimal concentration, while another highly metastatic cell line, WM266-4, demonstrated measurably lower binding (0.43 ± 0.02), and the low metastatic potential A375 cells showed the lowest binding (0.25 ± 0.02) (*p* < 0.01) (Fig. 1b).

#### JM1-24-3 interacts with the glycosylated MUC18 protein expressed on melanoma cells

To identify the JM1-24-3 target on melanoma cells, lysates of A2058, WM266-4 and A375 (25μg) were immunoprecipitated individually with 1μg each of JM1-24-3 and other irrelevant Abs followed by SDS-PAGE. One stronger band at 135kD alone was observed on probing with the same JM1-24-3 mAb, while no band was observed with other irrelevant antibodies. PBMC served as lysate control and β-actin (42kD) served as loading control (IP shown in Supplementary Fig. S1). Further, for mass spectrometry analysis, WM266-4 cells (100 μg) were immunoprecipitated with JM1-24-3 mAb (10 μg) alone. Due to increased lysate loading, two bands (one stronger band at 135kD and one faint band at 110kD) were identified in SDS-PAGE Coomassie staining (Fig. 1c). These bands were individually analyzed with mass spectrometry (MS) (Fig. 1d), which suggested that both bands matched the molecular weights of the MUC18 protein as reported previously [10]. We assumed that the two bands observed with two different molecular weights could be because of partial reduction of the MUC18 molecule due to protein denaturation and/or the presence of glycoproteins, rather than from dimer disassociation. Subsequently, the interaction between JM1-24-3 and its target was measured with Octet RED384 (ForteBio, LLC, Fremont, CA) on flow-through Bio-Layer Interferometry (BLI) chips with an average affinity constant K_D_ = 1.60E-09.

### Identifying the carbohydrate moieties and defining the conformational epitope of MUC18

#### Interaction of carbohydrate moieties in the functional MUC18 epitope

**Fig. 2:**
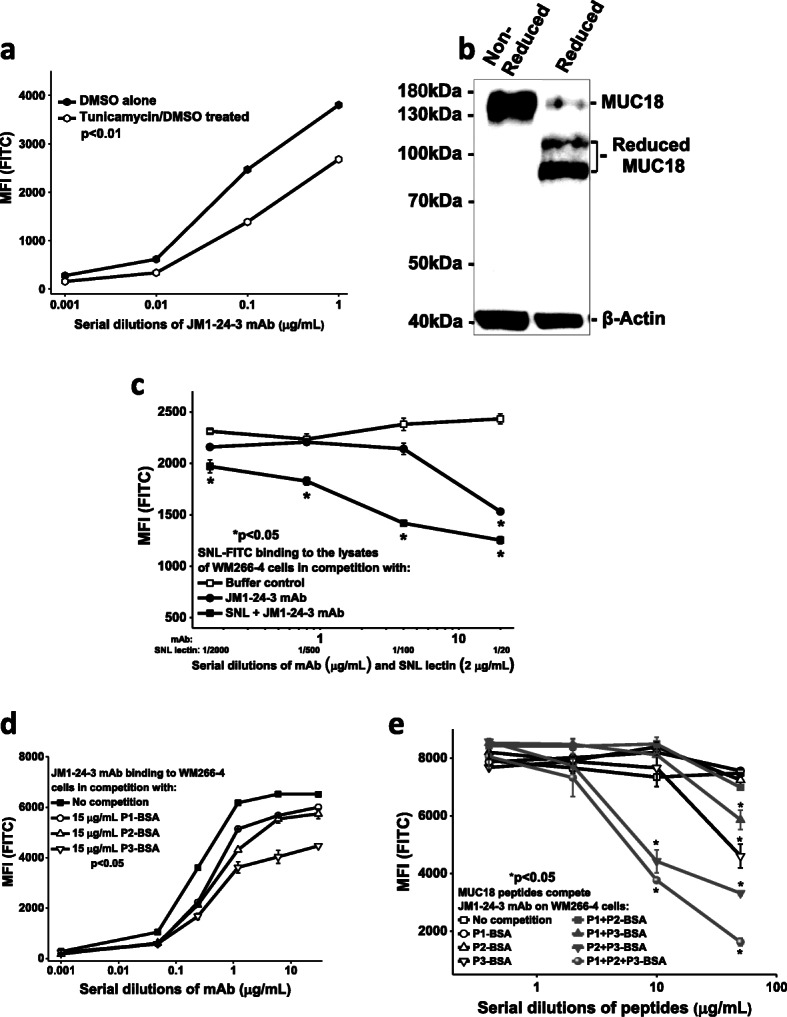
Identifying the carbohydrate moiety (ies) and defining the conformational epitope. *JM1-24-3 interacts with the glycosylated MUC18 protein expressed on the cell surface of melanoma cells* Treatment of WM266-4 cells with tunicamycin significantly reduced JM1-24-3 binding with MUC18 as shown by FACS (*p* < 0.01) **a** and showed reduced forms of MUC18 (110 and 90 kDa) by WB **b**. FLISA competition assay with WM266-4 cell membrane fraction showed that JM1-24-3 binding to MUC18 glycoprotein is significantly competed by lectin SNL **c**. *JM1-24-3 binds to the epitopes of MUC18 on melanoma cell surface* JM1-24-3 binding with WM266-4 is competitively reduced by MUC18 binding peptides, P1-BSA, P2-BSA and P3-BSA individually as shown by serial dilutions of Ab **d** or in combination **e** as shown by serial dilutions of peptides by FACS. The competitive interference achieved with combination of three peptides was greater than that achieved with the individual peptides

To evaluate the potential functional importance of the carbohydrate moieties in MUC18 and JM1-24-3 interaction sites, tunicamycin was used (Fig. 2). Tunicamycin treatment showed significant reduction (40.7 ± 7.47%; *p* < 0.01) in binding of JM1-24-3 to WM266-4 by FACS assay (Fig. 2a). These results suggested that tunicamycin partially digested the carbohydrates in N-linked glycosylation sites resulting in reduction of JM1-24-3 binding as demonstrated in Western blot (Fig. 2b). On tunicamycin treatment, MUC18 showed reduced forms of approximately 120 kDa and 90 kDa.

We also investigated the carbohydrate moiety (ies) of MUC18 using FLISA to elucidate key asparagine sites of N-linked glycosylation and evaluated the potential role of carbohydrate chains in the MUC18 epitope interacting with JM1-24-3. The binding of FITC conjugated SNL lectin to MUC18 on WM266-4 and competition with JM1-24-3 or SNL plus JM1-24-3 were quantified by MFI (Fig. 2c). In parallel experiments, JM1-24-3 did not compete with DSA-FITC, LCA-FITC or WGA-FITC (Supplementary Fig. S2A) for binding to the lysates of A375 cells under identical conditions in FLISA. SNL binds preferentially to sialic acid attached to terminal galactose in a-2,6 and to a lesser degree, a-2,3 linkage.

#### JM1-24-3 specifically interacts with a conformational epitope of MUC18

The epitope of MUC18 interacting with JM1-24-3 was inferred to represent a conformational, N-linked glycosylation site, and the glycoprotein was determined to most likely be the lectin SNL. We next evaluated JM1-24-3 binding to the epitope(s) of MUC18 through oligopeptide microarray (epitope mapping on biochips). The 8-mer and 6-mer tiling oligopeptides with one amino acid resolution derived from human MUC18 protein sequence were synthesized in situ (in triplicates) on microarray chip(s) and JM1-24-3 binding signals (MFI) were detected using anti-mouse IgG Alexa-647 secondary antibody. The binding peaks were analyzed using ArrayPro data processing software (Supplementary Fig. S2B). Reproducible results from this epitope mapping demonstrated binding peptides as peaks (signals) that aligned with specific MUC18 amino acid sequences (Supplementary Fig. S2C). Since the binding sites were sporadically spaced with different signal strength a conformational epitope/binding site for JM1-24-3 was suggested, with involvement of multiple MUC18 asparagine (N, Asn) residues.

Further, we validated JM1-24-3 binding to MUC18 using the three peptides selected from the identified epitope binding regions and showed their binding was competitively reduced by each of the MUC18 peptides (BSA conjugated P1-BSA, P2-BSA and P3-BSA), although to different degrees (Fig. 2d). To further confirm that these three peptides in combination could interfere with JM1-24-3 binding to the epitope of MUC18 on melanoma cells, 3 μg/mL of JM1-24-3 bound to WM266-4 cells was subjected to individual and combinatorial binding competition using serial dilutions of selected peptides (Fig. 2e). The competitive interference achieved with two or three peptides in combination was greater than that achieved with the peptides individually.

In order to evaluate the specificity of JM1-24-3 against MUC18, we compared the binding characteristics of JM1-24-3 with two commercially available antibodies directed against MUC18: mouse anti-human MUC18 mAb and goat polyclonal Ab together with an isotype control irrelevant mAb and goat serum (IgG). Representative flow cytometric analyses of MUC18 expression on the surface of WM266-4 cells is shown (Supplementary Fig. S2D). JM1-24-3 had a binding potency similar to that of the mouse anti-human MUC18 mAb. Similar results were obtained with another form of MUC18, a recombinant human MCAM protein (γHuMCAM), in indirect ELISA analysis (Supplementary Fig. S2E). We further tested the binding motif and potency of HRP-conjugated JM1-24-3 binding to γHuMCAM and WM266-4 lysate on ELISA, which were individually subjected to competition by JM1-24-3 (self-competition), mouse anti-human MUC18 mAb, irrelevant mAb and BSA/PBS buffer (Supplementary Fig. S2F). HRP-conjugated JM1-24-3 could be competed, to different extents, by JM1-24-3 (self-competition) and mouse anti-human MUC18 mAb in binding to both the γHuMCAM and WM266-4 lysates. These results demonstrated that JM1-24-3 had higher binding potency than the commercial mouse anti-human MUC18 mAb, and that these two mAbs did not share the same binding site on MUC18. In summary, the binding patterns for the two mAbs were similar in FACS and indirect ELISA, but not in competition ELISA and WB, suggesting they shared part, but not all, of their MUC18 binding epitope.

### JM1-24-3 binding to MUC18 glycoprotein on melanoma cell surface activates downstream signal transduction pathway involved in cell growth and proliferation

**Fig. 3:**
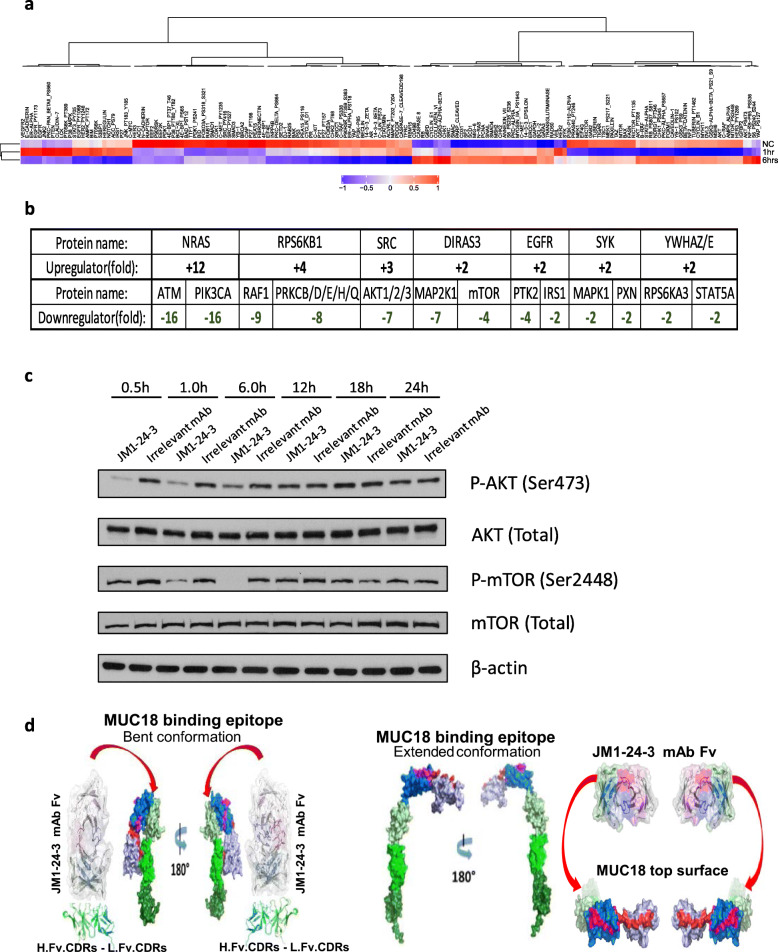
JM1-24-3 binds to MUC18 on melanoma cells and stimulates downstream signaling pathways in RPPA and computational models. **a** Heat maps illustrating the differences in the expression of proteins in WM266-4 cell lysates with 1 h/6 h/no treatment with JM1-24-3 as analyzed by RPPA. **b** Fold changes for up- or down-regulated proteins in RPPA as analyzed with the IPA software is shown. **c** WM266-4 cells treated with JM1-24-3/irrelevant mAb/PBS at different time points (30 min-24 h) showed by WB that p-AKT (Ser473) and p-mTOR (Ser2448) had time-dependent reduction in phosphorylation until 6 h, while both total AKT and mTOR remained unchanged and β-actin served as loading control. **d** Structural models showing the conformation of the MUC18 epitope, marked as pink, blue and red moieties, and relative binding of the heavy-chain, light-chain Fv peptides of JM1-24-3 to the flank and near side of the “bent” conformation compared to binding of the single-chain variable fragment (scFv) of JM1-24-3 to the top surface of MUC18 molecule when in its “extended” conformation

We sought to identify targets and downstream signaling events resulting from the binding of JM1-24-3 to MUC18 by RPPA. Both full length JM1-24-3 and its F (ab’)_2_ fragments (to avoid the background caused by the secondary goat anti-mouse IgG Fc pAb-FITC) reacted with MUC18 expressed on melanoma cells to induce down-stream signaling in tissue culture. WM266-4 cells were treated with full-length JM1-24-3 at two time points (1 h and 6 h) in tissue culture; the lysates from each time point were individually analyzed by RPPA. The heat maps in Fig. 3a demonstrated that approximately 150 signaling proteins were differentially expressed following JM1-24-3 treatment. By comparing with cell lysate without any treatment, several clusters of proteins showed elevated expression levels and a few others tended to have lower expression levels upon JM1-24-3 treatment. JM1-24-3 F (ab’)_2_ fragments revealed similar expression pattern in RPPA assay as well (data not shown). RPPA data was analyzed using IPA software (Fig. 3b). After JM1-24-3 interacted with MUC18 on WM266-4 cell surface, NRAS, RPS6Kβ1, and SRC were up-regulated, whereas ATM, PIK3CA, RAF1, PRKCB/D/E/H/Q, AKT1/2/3, MAP 2 K1, mTOR and PTK2 were down-regulated. To validate these RPPA data, WM266-4 cells were incubated with JM1-24-3 for 30 min-24 h. Time-dependent reduction in p-AKT (Ser473) and p-mTOR (Ser2448) was observed until 6 h, while total AKT and total mTOR remained unchanged (Fig. 3c).

The JM1-24-3 full-length mAb and F (ab’)_2_ treatment RPPA data together with IPA analysis revealed that JM1-24-3 binding to MUC18 initiated signaling in fundamentally important canonical cancer pathways, including PI3K/AKT and neuregulin; the top associated upstream regulators were TP53, MYC, ESR1 and others; the most important molecular and cellular functions impacted were those associated with cell death and survival, cellular development, and cellular growth and proliferation (Supplementary Fig. S3A).

Through a combination of structural investigations and computational modeling analyses, we defined molecular interactions between JM1-24-3 and its conformational epitope on MUC18. Homology structural modeling results are illustrated in Fig. 3d. We focused on MUC18 conformational changes predicted to result from exposure of the binding epitope to the JM1-24-3 antibody. We assumed that there were multiple confirmations of MUC18 on the cell surface (as for the majority of Ig superfamily adhesion molecules), and that the” bent” form of the MUC18 molecule, which buried the JM1-24-3 epitope, was the preferred, lower energy conformation since it had both less surface solvent exposure as well as more interaction forces. Upon binding of the antibody to the conformational epitope, there was a dynamic transition from the “bent” form to the extended form, which we presumed resulted in downstream signal transduction.

### JM1-24-3 inhibits melanoma cell growth and blocks Cancer cell migration and invasion by neutralization of MUC18

**Fig. 4:**
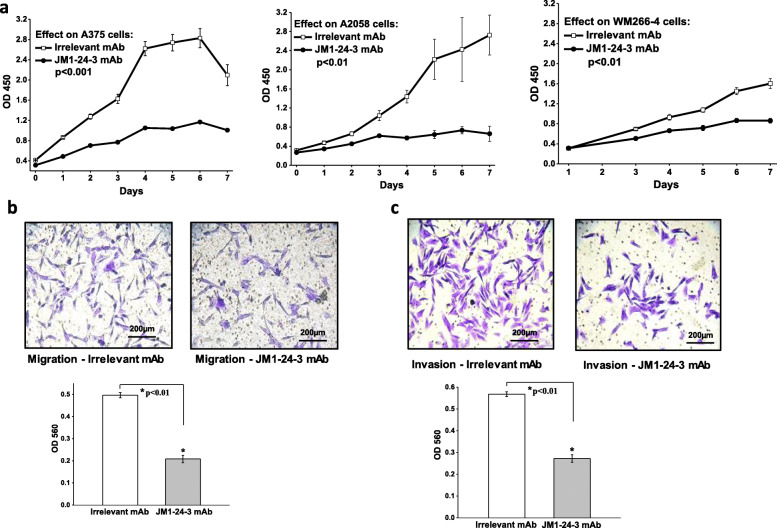
JM1-24-3 inhibits the proliferation, migration and invasion of melanoma cells **a** Treatment with JM1-24-3 (150 μg/mL) for seven days showed significant reduction in proliferation in A375 (52%), A2058 (76%) and WM266-4 (46%) cells as compared to irrelevant mAb treatment (*p* < 0.01). Colorimetric assays on the **b** migration of the WM266-4 cells treated with JM1-24-3 (150 μg/ml) showed significant reduction in migration (58%) and in **c** invasion assays (52%)

Based on RPPA data, we further performed cell based in vitro assays to evaluate the ability of JM1-24-3 to inhibit melanoma cell growth and proliferation. Proliferation assay showed that JM1-24-3 could significantly inhibit the proliferation of all three melanoma cell lines A375, A2058 and WM266-4 (Fig. 4a) (*p* < 0.01). Migration (Fig. 4b) and invasion (Fig. 4c) assays showed that JM1-24-3 had significant potency and efficacy in inhibition of migration and invasion of WM266-4 cells (*p* < 0.01). Similar effects were observed with A375 cells also (Supplementary Fig. S4A-B).

### JM1-24-3 inhibits melanoma tumor growth and metastasis in mouse Xenograft models

**Fig. 5:**
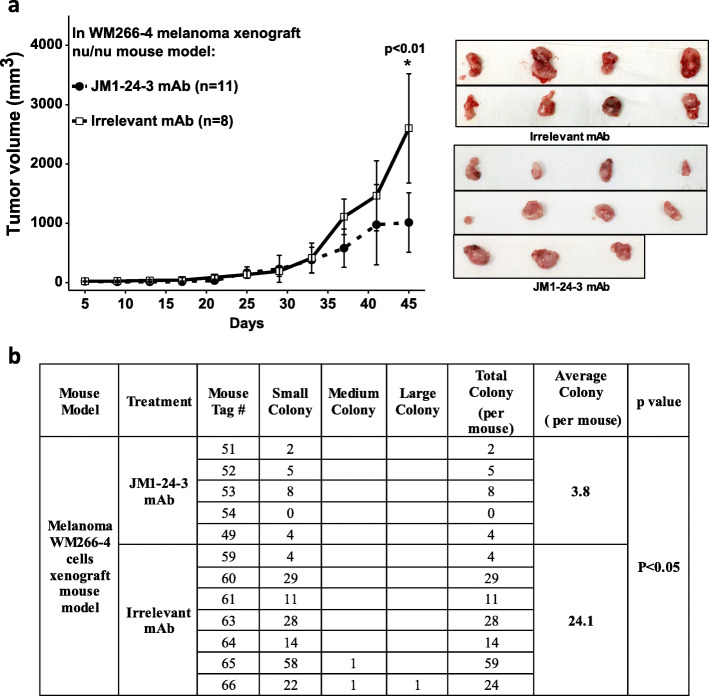
JM1-24-3 inhibited melanoma tumor growth and reduced lung metastasis in xenograft athymic nude mice. **a** Sub-cutaneous tumors were developed with WM266-4 cells on athymic nude mice and treated with JM1-24-3 (*n* = 11) or with irrelevant mAb (*n* = 8) (6 mg/kg body wt/i.p.) twice a week and tumor volume was measured every 4 days until day 45. Treatment with JM1-24-3 showed significant reduction in tumor volume (46.9 ± 11.8%; *p* < 0.01). **b** Pretreatment with JM1-24-3 (*n* = 5) or irrelevant mAb (*n* = 7) (6 mg/kg body wt/i.p/twice per week) was done one day before tail vein injection of WM266-4 cells on athymic nude mice followed by treatment till 45 days. All mice were sacrificed at day 45 and their lungs were harvested and stained with H&E and the number of metastatic tumor colonies was counted. Treatment with JM1-24-3 showed significantly fewer colonies (*p* < 0.05)

In athymic nude mice, WM266-4 cells (1 million) were injected subcutaneously; after 5 days of inoculation, mice were treated with either JM1-24-3 (*n* = 11) or with irrelevant mAb (*n* = 8) for 45 days (6 mg/kg body weight/i.p./twice per week), and tumor volume was measured with calipers every 4 days. No toxicity was observed, including no reduction in body weight on treatment with JM1-24-3 (data not shown). Tumors were excised and weighed at the end of the experiment. Treatment with JM1-24-3 showed significant reduction in tumor volume (46.8 ± 11.8; *p* < 0.01) as compared to irrelevant mAb treatment (Fig. 5a).

MUC18 expression has been reported to promote hematogenous lung metastasis [11]. We therefore evaluated whether JM1-24-3 could prevent or reduce melanoma lung metastasis in athymic nude mouse xenograft model. Pretreatment with JM1-24-3 (*n* = 5) or irrelevant mAb (*n* = 7) was done 1 day before tail vein injection of WM266-4 cells (6 mg/kg body weight/i.p./twice per week). All mice were sacrificed at day 45 and their lungs were harvested and stained with H&E (Supplementary Fig. S5) and the number of metastatic tumor colonies was counted and grouped to small, medium and large according to their sizes. The group treated with JM1-24-3 had significantly fewer colonies than the group treated with irrelevant mAb (*p* < 0.05) (Fig. 5b).

### The expression levels of MUC18 in melanoma have clinical significance

**Fig. 6:**
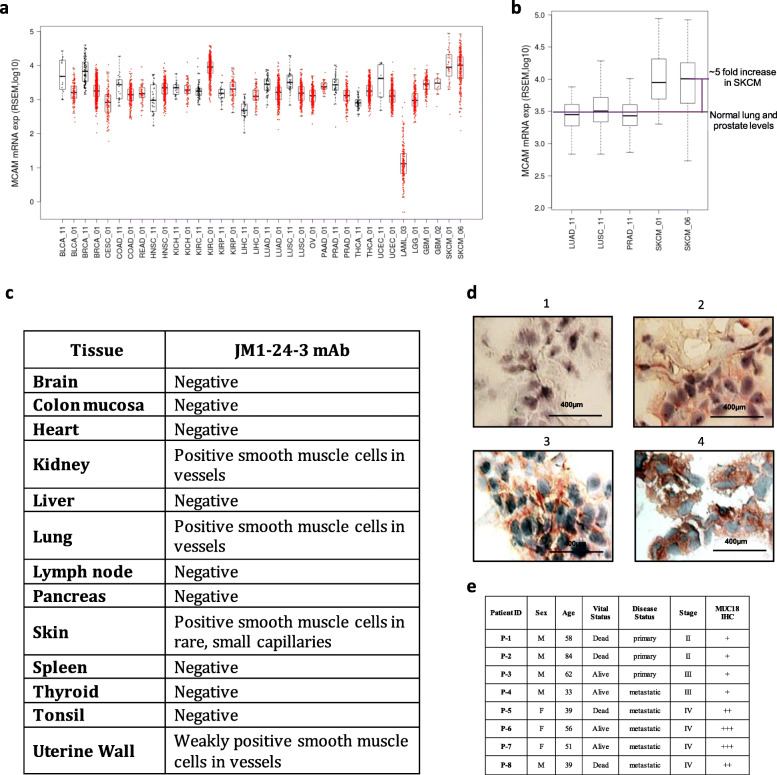
The expression levels of MUC18 in cancers have clinical significance. **a** The copy numbers of MUC18 mRNA were analyzed across a variety of cancers (red color) and normal tissues (black color) in TCGA cohort studies. The MUC18 gene expression was elevated in melanoma (SKCM) and renal cell carcinoma (KIRC). **b** The MUC18 gene expression level in melanoma (SKCM) was five times as much as that in normal lung and prostate tissues. **c** IHC of normal tissue microarray with JM1-24-3, showed positive staining on smooth muscle cells in small vessels of kidney, lung and skin, but not on vessels larger than small capillaries, while other normal tissues were negative. **d** MUC18 IHC images with JM1-24-3 showing variable staining intensity from negative (upper left panel) to strong positive (lower right panel) on melanoma patient tissue slides. **e** Staining intensity correlation of eight melanoma patients showed that metastatic melanoma patients had higher intensity staining of MUC18 with JM1-24-3 mAb. Also, all metastatic patients (5/5) showed stronger intensity

MUC18 mRNA copy numbers were analyzed across a variety of cancers and normal tissues using TCGA dataset and were found to be elevated in both melanoma (SKCM) and renal cell carcinoma (KIRC) (Fig. 6a). Focused comparison using normal lung and prostate tissues versus SKCM illustrated an approximately 5-fold increase of MUC18 mRNA level in SKCM compared to these other tissues (Fig. 6b).

Using TMA IHC, we investigated multiple normal tissues for MUC18 expression as measured by JM1-24-3 binding. Some expression was seen on smooth muscle cells within small vessels in the kidney, lung, skin and uterine wall, but medium and larger sized vessels were uniformly negative, and all other normal tissues evaluated were also negative (Fig. 6c).

Using JM1-24-3 IHC staining, we investigated tumors from eight melanoma patients with various stages of disease. Representative results of IHC staining are shown in Fig. 6d and the IHC findings were correlated with the clinical status of the patients (Fig. 6e). Although the sample size is small, the results suggest higher expression of MUC18 on melanoma tissues from patients with metastatic melanoma. JM1-24-3 binding was compared with commercially available mouse anti-human MUC18 Ab on IHC on patient tissues. JM1-24-3 showed higher staining intensity and increased staining of cancer cells compared to commercial Ab at the same dilution (Supplementary Fig. S6).

Comparing the binding signals by JM1-24-3 to the surface of various cancer cell lines, melanoma cell lines A2058 and WM266-4 showed the strongest signal, the gastric cancer (GC) cell line MKN45 and EC-RF24 had medium binding signals; triple-negative breast cancer (TNBC) cell lines MDA-MB-231 and MDA-MB-468 and the GC cell line BGC823 had weak binding signals (Supplementary Fig. S7), whereas human PBMC had no detectable signal.

## Discussion

In the current study, a novel strategy of live cell immunization and live cell high-throughput screening by FACS analysis was used for the development and screening of antibodies directed against antigens on the surface of metastatic melanoma tumor cells. Multiple protein-, peptide- and cell-based assays were used to define JM1-24-3 as a specific antibody binding to an identified conformational epitope on its target on cancer cells. Mass spectrometry analysis suggested the target as MUC18 (CD146).

Circulating MUC18 is a promising melanoma biomarker; circulating levels are significantly associated with poor prognosis and death [12, 13]. MUC18 interacts with several receptor proteins, including Calprotectin (S100 calcium-binding protein A8/A9, S100A8/A9), heparin sulfate, Toll Like Receptor 4 (TLR4) and Receptor for Advanced Glycation End products (RAGE) [14]. Thus, MUC18 may play role in multiple processes including inflammation, cell differentiation, adhesion, tumorigenesis, migration, invasion, angiogenesis, and metastasis [15]. MUC18 has been shown to induce translational initiation and transcriptional activation of c-Jun/c-Fos in hepatocellular carcinoma [16] and has been suggested as a potential therapeutic target in malignant rhabdoid tumors through induction of apoptosis by inactivating protein kinase B (PKB) or the serine/threonine-specific kinase (AKT) signaling pathway [17].

Evaluation of glycosylation of MUC18 determined that sugar residues were involved in the structure of the molecule, and that functional interactions between JM1-24-3 and the conformational epitope of MUC18 were dependent on glycosylation. Recent literature suggests that MUC18 can be glycosylated via N-acetyl-glucosaminyltransferease III and V without a role in migration [18] or could be glycosylated and stabilized by β-1,3-galactosyl-O-glycosyl-glycoprotein β-1,6-N-acetylglucosaminyltransferase 3 (GCNT3) playing a major role in melanoma migration and invasion [19]. Thus, involvement of glycosylated conformational epitope in the function of MUC18 could therefore have implications in melanoma progression or metastasis.

Importantly, JM1-24-3 was capable of binding to MUC18 expressed on the melanoma cell surface, subsequently inducing downstream signaling pathways and further inhibition of cell growth and metastasis. Mechanistic investigations demonstrated induction of multiple changes in downstream signaling pathways following binding of JM1-24-3 to MUC18. Furthermore, JM1-24-3 demonstrated only weak, patchy binding to smooth muscle cells in small vessels of the kidney, lymph nodes and skin, and was otherwise unreactive across a cross-section of normal tissues. Hence, this preliminary data suggests that targeting MUC18 via the conformational epitope identified by JM1-24-3 may have limited toxicity. Finally, in addition to confirming that MUC18 was highly expressed on melanoma tumors, especially metastatic tumors, we identified significant MUC18 expression in several other cancers, including those of the gastrointestinal tract and TNBC.

Although a small number of potentially therapeutic antibodies have been recently developed against MUC18 [20], the specific advantage of JM1-24-3 is that our studies document that it recognizes a conformational epitope of MUC18 on the cancer cell surface, and in doing so alters downstream signaling pathways, resulting in reduction of associated tumor-promoting functions. While we cannot completely exclude the possibility that JM1-24-3 could have additional, functionally relevant melanoma targets, we submit that the data provided are strong evidence that JM1-24-3 recognizes a specific conformational epitope on MUC18, and in binding to that epitope induces downstream signaling events that mediate the metastatic phenotype, thus identifying the MUC18 conformational epitope as a promising therapeutic target potentially amenable to mAb treatment. Also, since MUC18 is expressed on the surface of other cancers, JM1-24-3 could be a useful therapeutic agent for therapy of other cancers, either alone or in combination with other agents.

## Conclusion

A novel mouse monoclonal antibody JM1-24-3, developed by melanoma live cells immunization, was identified to be directed against the conformational epitope of the cell surface antigen MUC18. JM1-24-3 blocked MUC18 downstream signaling pathways and cellular functions in vitro and in vivo. Thus, the MUC18 conformational epitope identified represents a promising therapeutic target, and the JM1-24-3 mAb might serve as the basis for a potential therapeutic agent.

## Supplementary information

**Additional file 1.**

## Data Availability

All data generated or analyzed during this study are included in this published article and its supplementary information files. Further information and requests for resources and reagents should also be directed to the corresponding authors.

## Abbreviations

HTS: High-throughput screening
MAb: Monoclonal antibody
MCAM: Melanoma cell adhesion molecule
CD146: Cluster of differentiation 146
METCAM/MelCAM: Metastatic melanoma CAM
IACUC: Institutional animal care and use committee
ATCC: American type culture collection
PBMC: Human peripheral blood mononuclear cells
DMEM: Dulbecco’s modified eagle’s medium
FFPE: Formalin-fixed paraffin embedded
TMA: Tissue micro arrays
s.c.: Subcutaneously
i.p.: Intraperitoneal
FACS: Fluorescence-activated cell sorting
MFI: Mean fluorescence intensity
DMSO: Dimethyl sulfoxide
WB: Western blot
SDS-PAGE: Sodium dodecyl sulfate polyacrylamide gel electrophoresis
ELISA: Enzyme-linked immunosorbent assay
FLISA: Fluorescence-linked immunosorbent assay
FITC: Fluorescein isothiocyanate
HRP: Horseradish peroxidase
Alexa Fluor 647: Far-red–fluorescent dye
SNL: *Sambucus nigra* lectin
DSA: *Datura stramonium* agglutinin
LCA: *Lens culinaris* agglutinin
WGA: Wheat germ agglutinin
IHC: Immunocytochemistry
IP: Immunoprecipitation
MS: Mass spectrometry
RPPA: Reverse phase protein array
IPA: Ingenuity pathway analysis
BLI: Bio-layer interferometry chips
Fc region: Fragment crystallizable region
Fab fragment: Fragment antigen-binding
*nu/nu*: Athymic nude mice
SD: Standard deviation
Calprotectin: S100 calcium-binding protein A8/A9, S100A8/A9
TLR4: Toll like receptor 4
RAGE: Receptor for advanced glycation end products
PKB: Protein kinase B
AKT: Serine/threonine-specific kinase
GCNT3: β-1,3-galactosyl-O-glycosyl-glycoprotein β-1,6-N-acetylglucosaminyltransferase 3
