# Supplementary material for: Characterization of novel neutralizing mouse monoclonal antibody JM1-24-3 developed against MUC18 in metastatic melanoma
Source: J Exp Clin Cancer Res. 2020 Dec 5;39:273. doi: 10.1186/s13046-020-01722-8 (PMC7718695; doi:10.1186/s13046-020-01722-8)
Supplement: Supplementary file 1 — Additional file 1. [file 13046_2020_1722_MOESM1_ESM.pptx]

## Slide 1
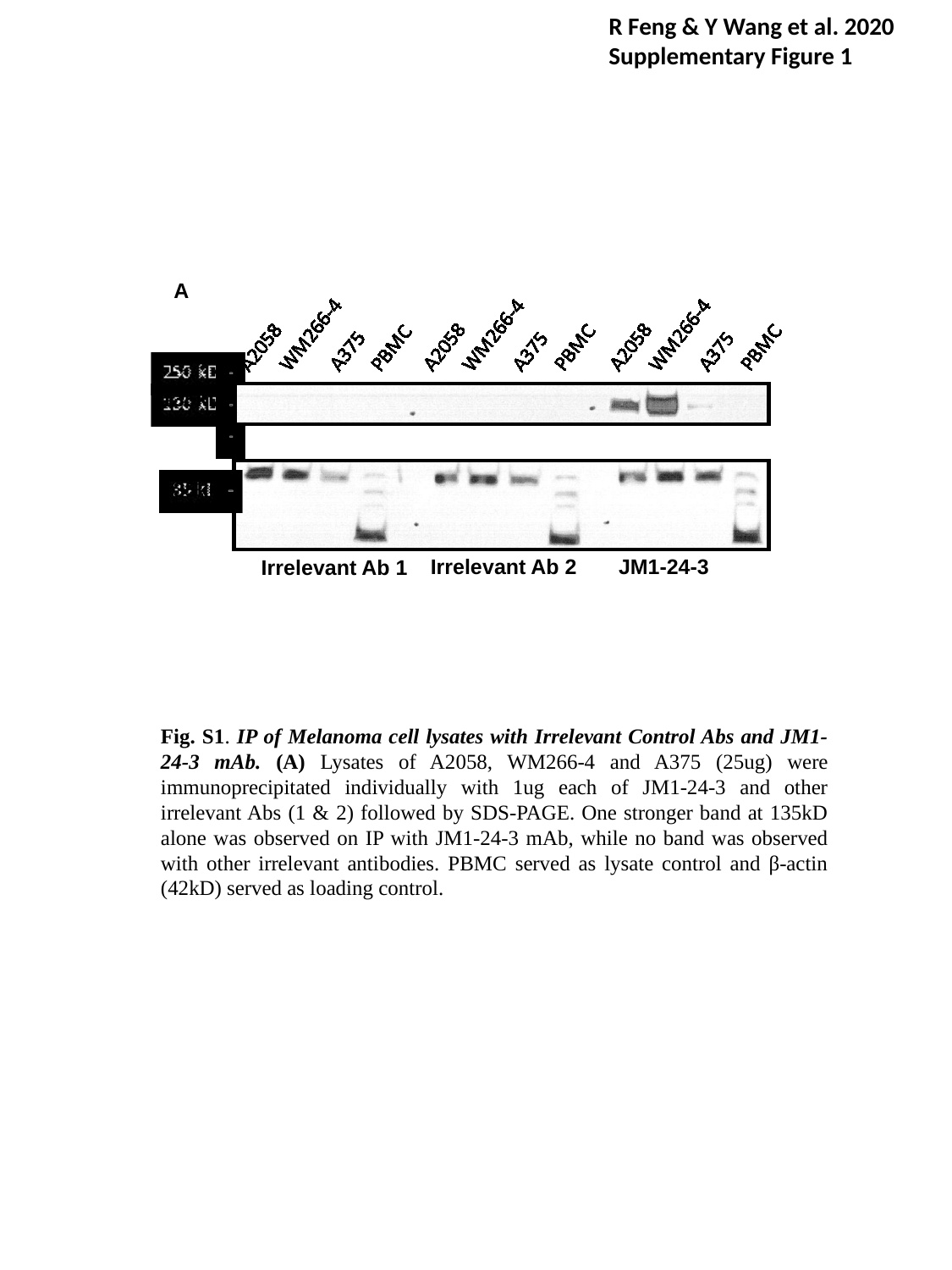

R Feng & Y Wang et al. 2020
Supplementary Figure 1
A
135kD
35kD
Irrelevant Ab 2
JM1-24-3
Irrelevant Ab 1
Fig. S1. IP of Melanoma cell lysates with Irrelevant Control Abs and JM1-24-3 mAb. (A) Lysates of A2058, WM266-4 and A375 (25ug) were immunoprecipitated individually with 1ug each of JM1-24-3 and other irrelevant Abs (1 & 2) followed by SDS-PAGE. One stronger band at 135kD alone was observed on IP with JM1-24-3 mAb, while no band was observed with other irrelevant antibodies. PBMC served as lysate control and β-actin (42kD) served as loading control.

## Slide 2
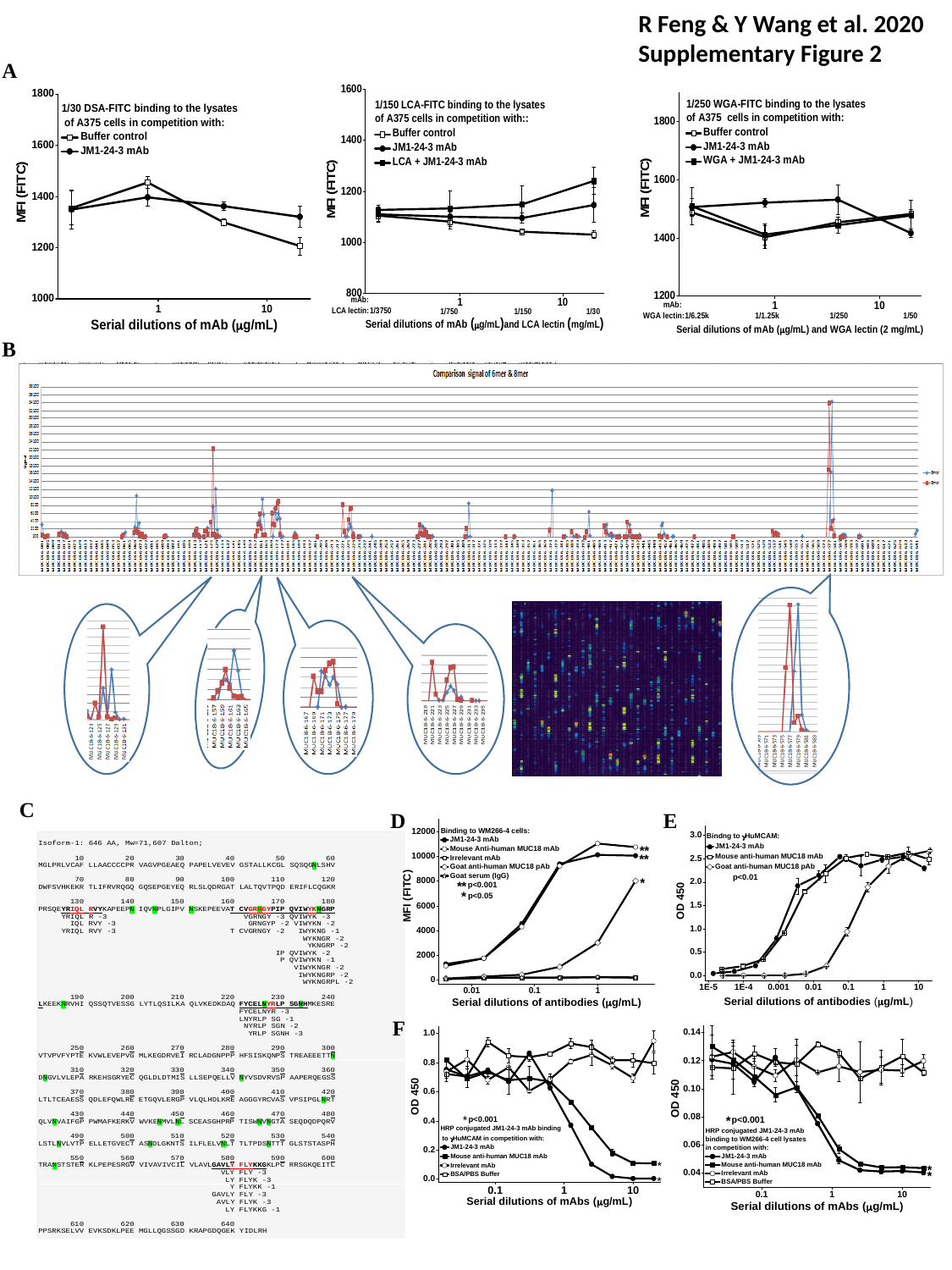

R Feng & Y Wang et al. 2020
Supplementary Figure 2
A
B
C
E
D
F

## Slide 3
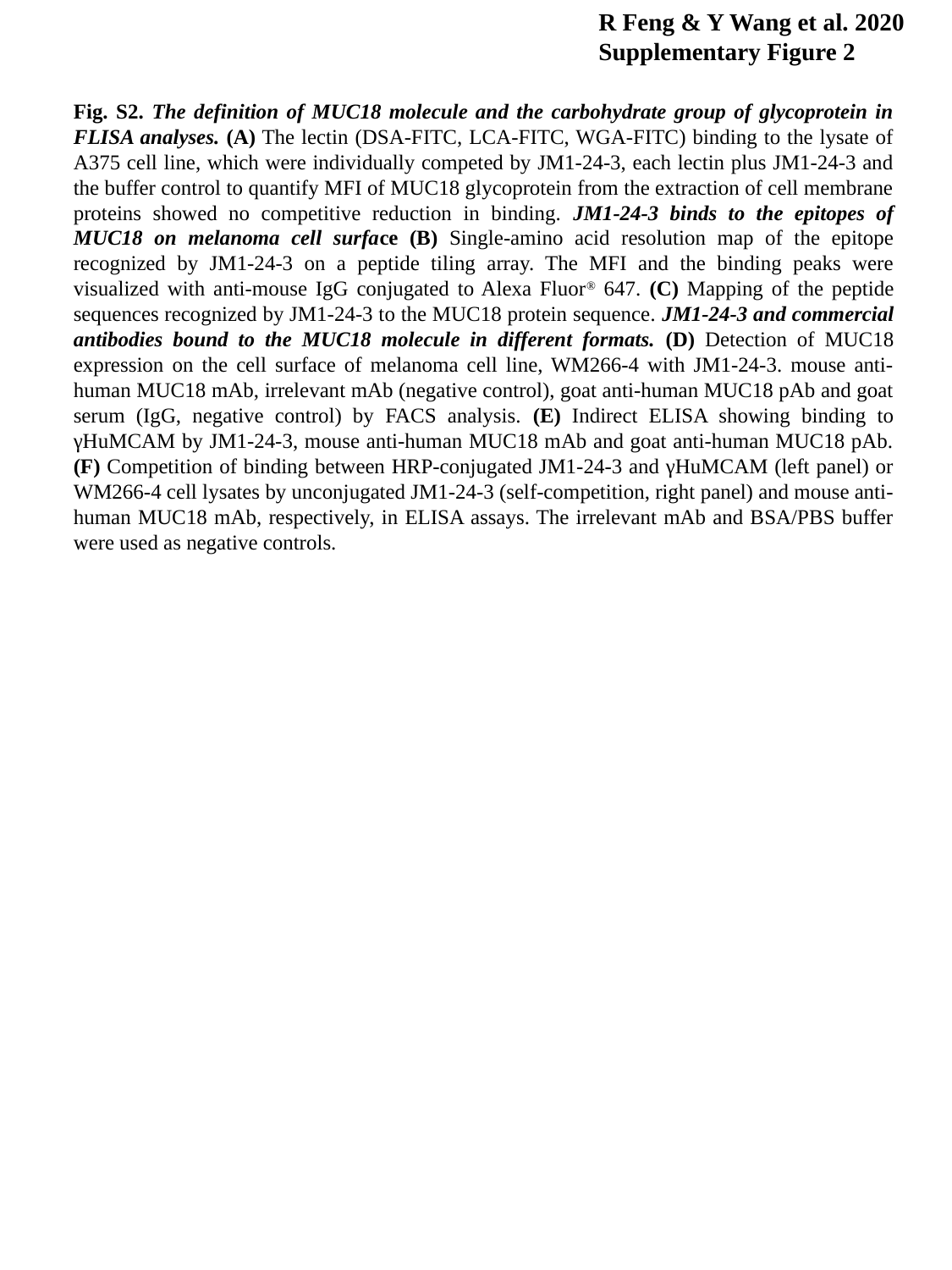

R Feng & Y Wang et al. 2020
Supplementary Figure 2
Fig. S2. The definition of MUC18 molecule and the carbohydrate group of glycoprotein in FLISA analyses. (A) The lectin (DSA-FITC, LCA-FITC, WGA-FITC) binding to the lysate of A375 cell line, which were individually competed by JM1-24-3, each lectin plus JM1-24-3 and the buffer control to quantify MFI of MUC18 glycoprotein from the extraction of cell membrane proteins showed no competitive reduction in binding. JM1-24-3 binds to the epitopes of MUC18 on melanoma cell surface (B) Single-amino acid resolution map of the epitope recognized by JM1-24-3 on a peptide tiling array. The MFI and the binding peaks were visualized with anti-mouse IgG conjugated to Alexa Fluor® 647. (C) Mapping of the peptide sequences recognized by JM1-24-3 to the MUC18 protein sequence. JM1-24-3 and commercial antibodies bound to the MUC18 molecule in different formats. (D) Detection of MUC18 expression on the cell surface of melanoma cell line, WM266-4 with JM1-24-3. mouse anti-human MUC18 mAb, irrelevant mAb (negative control), goat anti-human MUC18 pAb and goat serum (IgG, negative control) by FACS analysis. (E) Indirect ELISA showing binding to γHuMCAM by JM1-24-3, mouse anti-human MUC18 mAb and goat anti-human MUC18 pAb. (F) Competition of binding between HRP-conjugated JM1-24-3 and γHuMCAM (left panel) or WM266-4 cell lysates by unconjugated JM1-24-3 (self-competition, right panel) and mouse anti-human MUC18 mAb, respectively, in ELISA assays. The irrelevant mAb and BSA/PBS buffer were used as negative controls.

## Slide 4
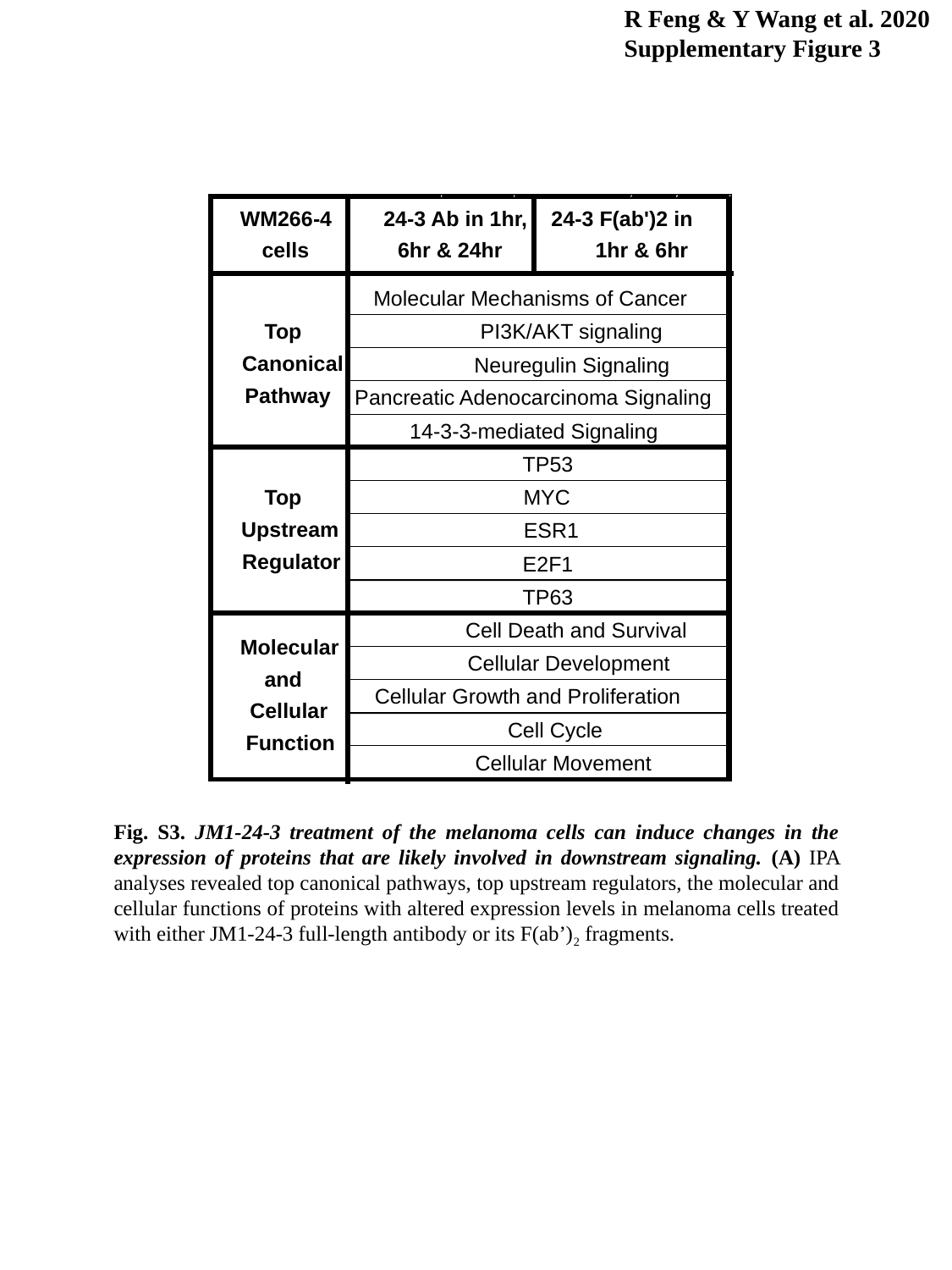

R Feng & Y Wang et al. 2020
Supplementary Figure 3
WM266-4
24-3 Ab in 1hr,
24-3 F(ab')2 in
cells
6hr & 24hr
1hr & 6hr
Molecular Mechanisms of Cancer
Top
PI3K/AKT signaling
Canonical
Neuregulin Signaling
Pathway
Pancreatic Adenocarcinoma Signaling
14-3-3-mediated Signaling
TP53
Top
MYC
Upstream
ESR1
Regulator
E2F1
TP63
Cell Death and Survival
Molecular
Cellular Development
and
Cellular Growth and Proliferation
Cellular
Cell Cycle
Function
Cellular Movement
Fig. S3. JM1-24-3 treatment of the melanoma cells can induce changes in the expression of proteins that are likely involved in downstream signaling. (A) IPA analyses revealed top canonical pathways, top upstream regulators, the molecular and cellular functions of proteins with altered expression levels in melanoma cells treated with either JM1-24-3 full-length antibody or its F(ab’)2 fragments.

## Slide 5
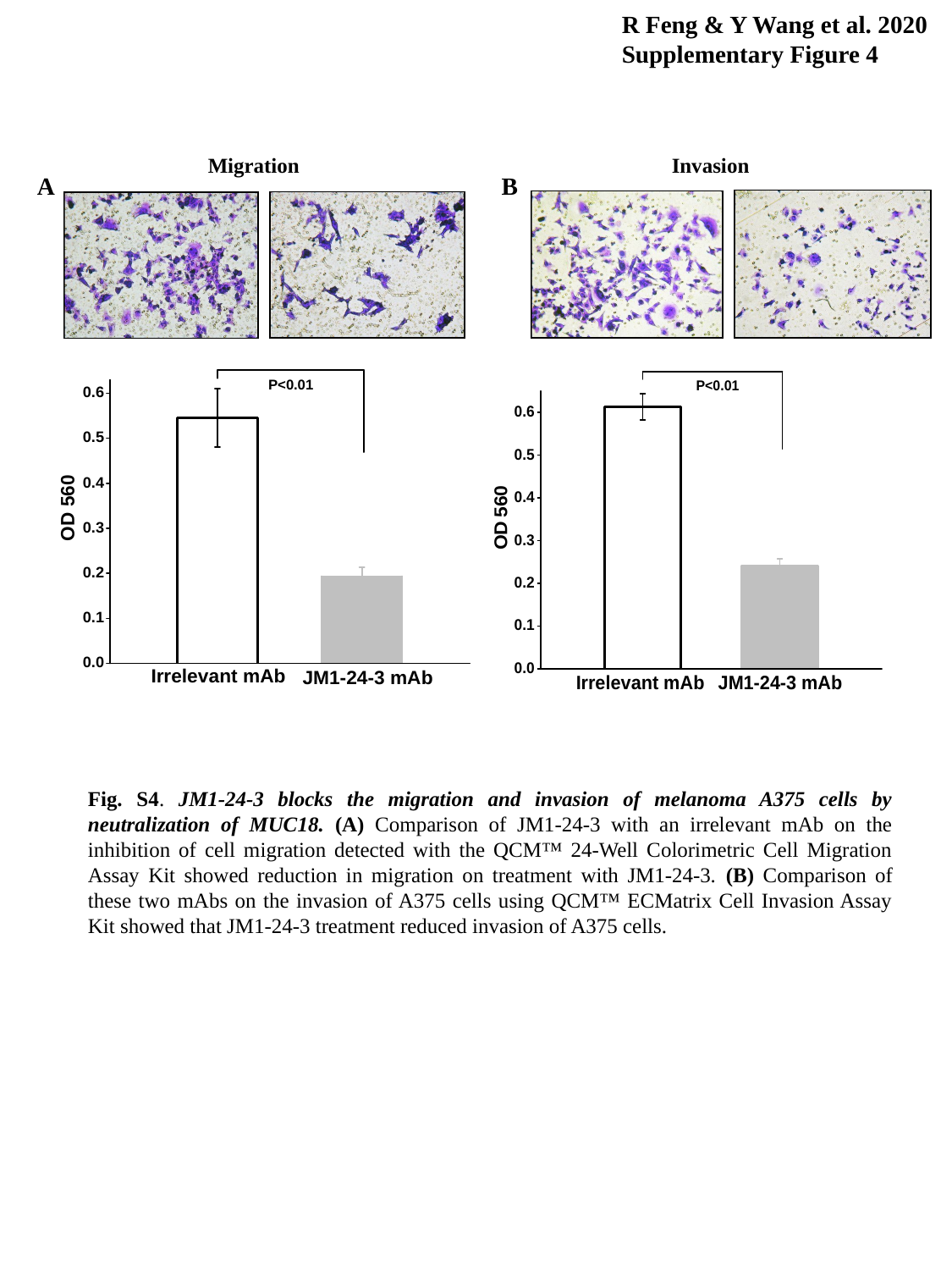

R Feng & Y Wang et al. 2020
Supplementary Figure 4
Migration
Invasion
A
B
Fig. S4. JM1-24-3 blocks the migration and invasion of melanoma A375 cells by neutralization of MUC18. (A) Comparison of JM1-24-3 with an irrelevant mAb on the inhibition of cell migration detected with the QCM™ 24-Well Colorimetric Cell Migration Assay Kit showed reduction in migration on treatment with JM1-24-3. (B) Comparison of these two mAbs on the invasion of A375 cells using QCM™ ECMatrix Cell Invasion Assay Kit showed that JM1-24-3 treatment reduced invasion of A375 cells.

## Slide 6
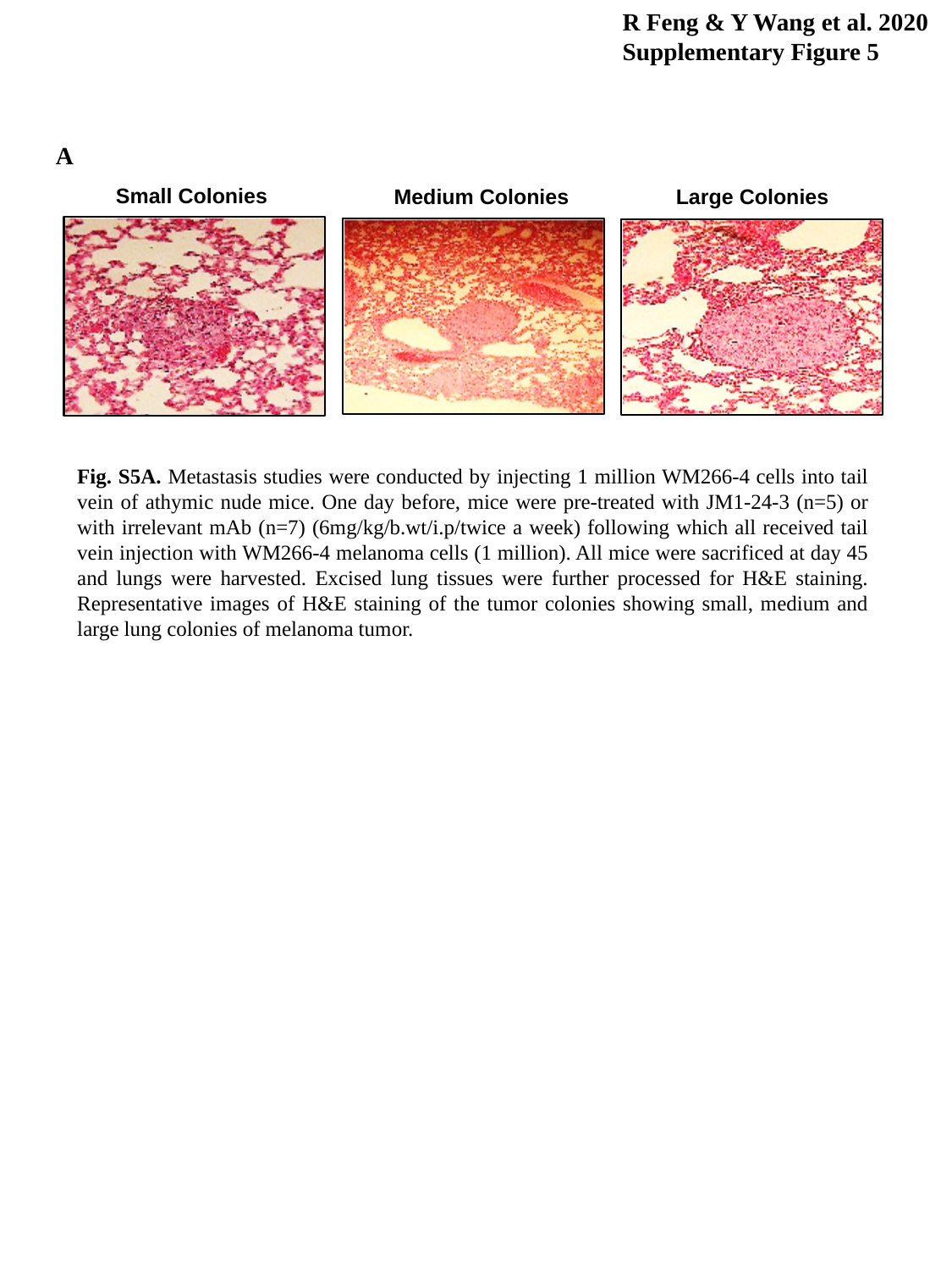

R Feng & Y Wang et al. 2020
Supplementary Figure 5
A
Small Colonies
Medium Colonies
Large Colonies
Fig. S5A. Metastasis studies were conducted by injecting 1 million WM266-4 cells into tail vein of athymic nude mice. One day before, mice were pre-treated with JM1-24-3 (n=5) or with irrelevant mAb (n=7) (6mg/kg/b.wt/i.p/twice a week) following which all received tail vein injection with WM266-4 melanoma cells (1 million). All mice were sacrificed at day 45 and lungs were harvested. Excised lung tissues were further processed for H&E staining. Representative images of H&E staining of the tumor colonies showing small, medium and large lung colonies of melanoma tumor.

## Slide 7
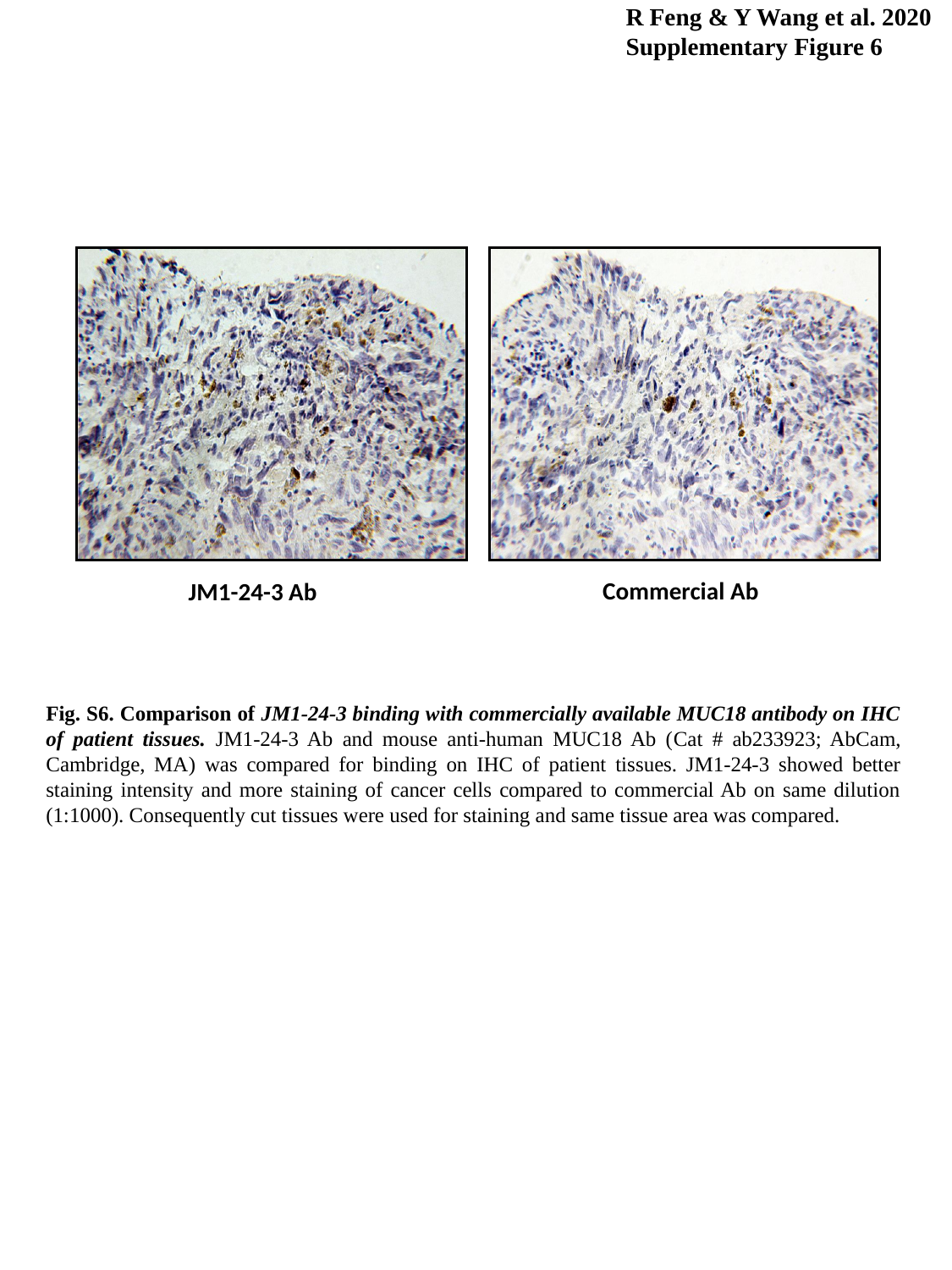

R Feng & Y Wang et al. 2020
Supplementary Figure 6
Commercial Ab
JM1-24-3 Ab
Fig. S6. Comparison of JM1-24-3 binding with commercially available MUC18 antibody on IHC of patient tissues. JM1-24-3 Ab and mouse anti-human MUC18 Ab (Cat # ab233923; AbCam, Cambridge, MA) was compared for binding on IHC of patient tissues. JM1-24-3 showed better staining intensity and more staining of cancer cells compared to commercial Ab on same dilution (1:1000). Consequently cut tissues were used for staining and same tissue area was compared.

## Slide 8
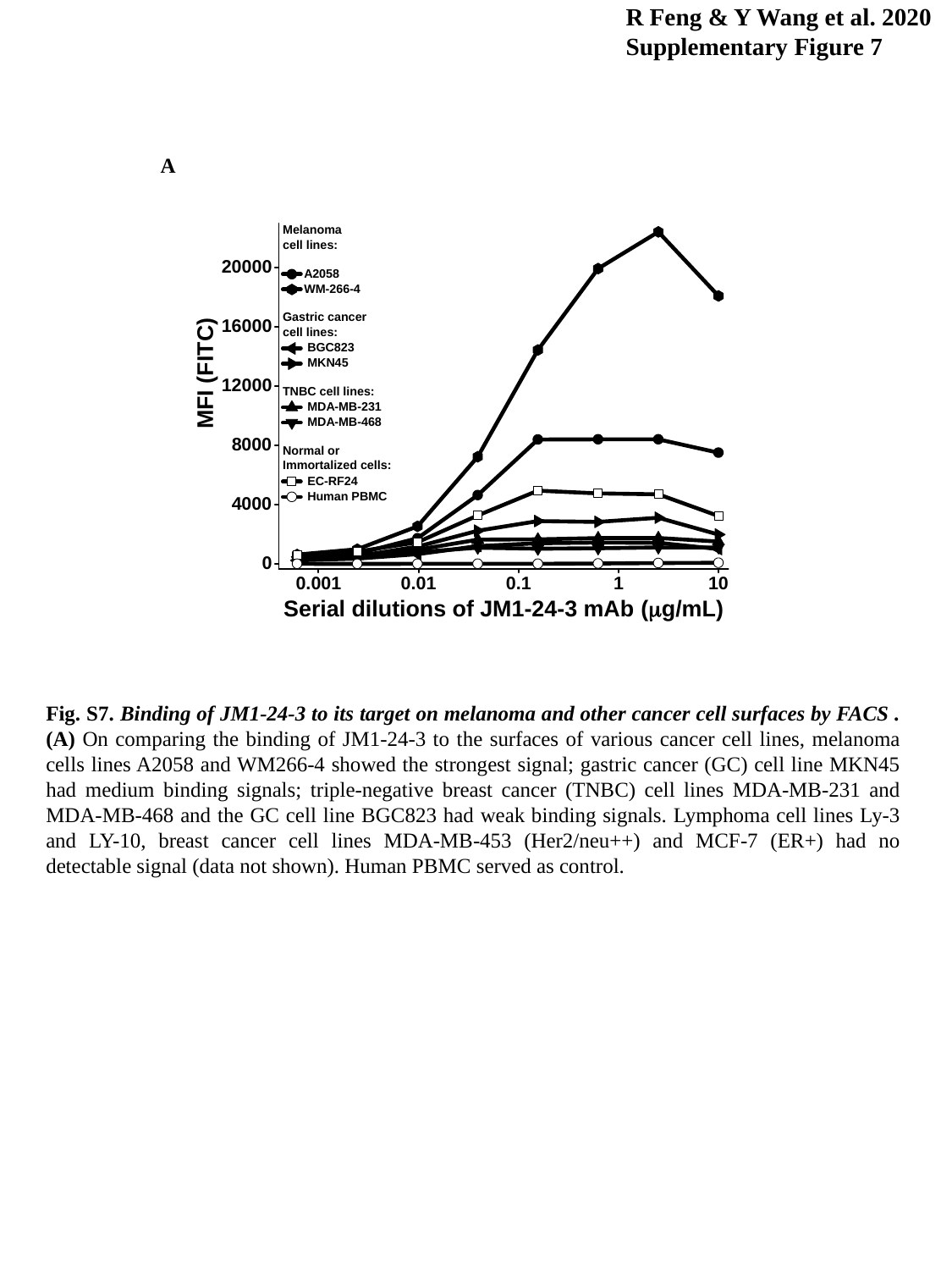

R Feng & Y Wang et al. 2020
Supplementary Figure 7
A
Fig. S7. Binding of JM1-24-3 to its target on melanoma and other cancer cell surfaces by FACS . (A) On comparing the binding of JM1-24-3 to the surfaces of various cancer cell lines, melanoma cells lines A2058 and WM266-4 showed the strongest signal; gastric cancer (GC) cell line MKN45 had medium binding signals; triple-negative breast cancer (TNBC) cell lines MDA-MB-231 and MDA-MB-468 and the GC cell line BGC823 had weak binding signals. Lymphoma cell lines Ly-3 and LY-10, breast cancer cell lines MDA-MB-453 (Her2/neu++) and MCF-7 (ER+) had no detectable signal (data not shown). Human PBMC served as control.
